# High risk and low uptake of pre‐exposure prophylaxis to prevent HIV acquisition in a national online sample of transgender men who have sex with men in the United States

**DOI:** 10.1002/jia2.25391

**Published:** 2019-09-19

**Authors:** Sari L Reisner, Chiara S Moore, Andrew Asquith, Dana J Pardee, Aaron Sarvet, Gal Mayer, Kenneth H Mayer

**Affiliations:** ^1^ The Fenway Institute Fenway Health Boston MA USA; ^2^ Department of Pediatrics Boston Children's Hospital Harvard Medical School Boston MA USA; ^3^ Department of Epidemiology Harvard T.H. Chan School of Public Health Boston MA USA; ^4^ Gilead Sciences, Inc. Foster City CA USA; ^5^ Department of Medicine Harvard Medical School Boston MA USA; ^6^ Global Population Health Harvard T.H. Chan School of Public Health Boston MA USA

**Keywords:** HIV, men who have sex with men (MSM), sexual and gender minorities, PrEP, social stigma, transgender persons

## Abstract

**Introduction:**

Trans masculine people who have sex with cisgender (“cis”) men (“trans MSM”) may be at‐risk for HIV infection when they have cis MSM partners or share needles for hormone or recreational drug injection. Limited data are available characterizing indications and uptake of pre‐exposure prophylaxis (PrEP) in trans MSM. The aim of this study was to assess PrEP indication and uptake as a means of primary HIV prevention for adult trans MSM in the U.S.

**Methods:**

Between November and December 2017, a national convenience sample of trans MSM in the U.S. (n = 857) was recruited using participatory methodologies and completed an online survey of demographics, HIV risk, PrEP, behavioural and psychosocial factors. Self‐reported receptive anal sex or frontal/vaginal sex (with or without a condom) with a cis male sex partner in past six months was an eligibility criterion. A multivariable logistic regression procedure was used to model PrEP indications (yes/no) per an interpretation of U.S. Centers of Disease Control and Prevention recommendations among those without HIV (n = 843).

**Results:**

The diverse sample was 4.9% Black; 22.1% Latinx ethnicity; 28.4% non‐binary gender identity; 32.6% gay‐identified; 82.7% on testosterone. Overall, 84.1% had heard of PrEP. Of these, 33.3% reported lifetime PrEP use (21.8% current and 11.5% past). Based on HIV behavioural risk profiles in the last six months, 55.2% of respondents had indications for PrEP. In a multivariable model, factors associated with PrEP indication included where met sex partners, not having sex exclusively with cismen, higher perceived HIV risk, greater number of partners and high cis male partner stigma (all *p *<* *0.05).

**Discussion:**

The majority of trans MSM in this sample had a PrEP indication. Stigma was associated with risk for HIV acquisition and represents a critical target for HIV biobehavioural prevention interventions for trans MSM, who appear to be underutilizing PrEP.

**Conclusions:**

Results from this study support the full inclusion of trans MSM in HIV biobehavioural prevention efforts. Public health interventions and programmes are needed to reach trans MSM that attend to general MSM risk factors as well as to vulnerabilities specific to trans MSM, including the context of stigma from cis male sexual partners.

## 1 Introduction

In the United States (U.S.), HIV infection remains a serious public health problem, particularly among key populations, such as men who have sex with men (MSM) 1. Transgender people, those who have a gender identity that differs from the sex assigned to them at birth, are also a population at elevated risk for HIV infection. Historically, HIV‐related research among transgender people has focused on transgender (“trans”) women, those assigned a male sex at birth who identify as women or another trans feminine gender identity 2, 3. Studies demonstrate that trans women are disproportionately affected by HIV infection relative to (“cis”) people 4 and have been a focus of HIV prevention interventions, including pre‐exposure prophylaxis (PrEP) to prevent HIV. Only recently, have researchers begun to evaluate HIV risk among trans masculine people, those assigned a female sex a birth who identify as men, transgender men, or another gender identity on the trans masculine spectrum 5, 6. Trans masculine individuals have often been erroneously thought to identify exclusively as heterosexual or to engage in sex with only cis women 7. However, trans masculine individuals engage in sexual activity with partners of many different genders, including cis males 8, 9, 10. Trans men who have sex with cis males (trans MSM) have been identified as a potentially vulnerable subgroup to HIV infection 11. Studies have commenced to document risks for HIV infection for trans MSM, including condomless receptive anal and/or frontal/vaginal sex with cis male partners 2. Trans MSM may be at‐risk for HIV infection when they have cis MSM partners or share needles for hormone or recreational drug injection. To the best of our knowledge, however, there have been no national studies conducted to understand and characterize HIV risks and prevention needs of trans MSM, including PrEP indication, in the U.S 11.

PrEP is a safe and effective method of HIV prevention 12. The first step in the PrEP care continuum is identifying individuals at highest risk for contracting HIV by assessing sexual history and other risk criteria 13. Currently, the Centers for Disease Control and Prevention (CDC) has PrEP indication guidelines for several populations in the HIV epidemic such as for MSM, heterosexuals and injection drug users (IDU) 14. PrEP use is clinically recommended for the individuals who meet the specified criteria. However, in the current absence of data about trans MSM HIV risks and vulnerabilities, it is not clear what criteria should be used to assess for PrEP indication in this group. Trans MSM who could be potential candidates for PrEP care, or behavioural HIV risk reduction generally, are likely being overlooked by medical providers due to the gap in research. To the best of our knowledge, only one small qualitative PrEP study of trans men has been published in the peer‐reviewed literature to date 15. This study found low levels of information about PrEP among trans men sampled and barriers to PrEP access, including financial costs. Research is needed to inform future PrEP indication guidelines and assess epidemiologic risks in trans MSM.

Some factors associated with HIV risk and PrEP indication among trans MSM may be similar to those for cis MSM 16. Trans MSM may face healthcare barriers, such as lack of health insurance, suboptimal access to HIV testing or delays in utilization of prevention services 17, 18, 19, 20, 21. Mental health conditions such as psychological distress and substance use behaviours including alcohol and illicit drug use may influence HIV risk behaviours 9, 22, 23, 24. Partner‐related factors may be relevant for considering PrEP for trans MSM, including non‐monogamous relationships and places where sexual partners are met 3, 19. HIV risk perception 25, 26 and internalized sexual minority stigma 27 have each been shown relevant for HIV prevention in cis MSM and may also be important to consider for trans MSM.

Yet trans MSM may have unique vulnerabilities that differ from cis MSM. Trans MSM are a stigmatized subgroup at the complex intersection of both gender and sexual minority statuses. Thus, dual exposure to gender and sexual minority socialization and stress pathways may increase risk of HIV in trans MSM 8. In addition to sexual minority stigma stressors 28, trans MSM may experience gender minority‐related stigma 29 exposures such as those resulting from having a non‐binary gender identity (identifying outside the traditional male‐female gender binary). Socialization stressors may be relevant, particularly for gay‐identified trans MSM who may experience gay community norms about sexual behaviours, sexual risk or other practices (e.g. HIV sero‐sorting) 19. Experiencing stigma from cis male sex partners may activate socialization and stress pathways around gender norms and HIV risk for trans MSM 8. For example, trans MSM may feel pressure from cis male sex partners to engage in risky sexual behaviours prioritizing validation of their gender identity as men 2, 15. Studies are needed to identify the specific vulnerabilities facing trans MSM for future interventional research, including PrEP service delivery.

To fill these research gaps, the current study sought to characterize PrEP awareness, uptake and indications in a sample of trans MSM in the U.S. using different adapted CDC algorithms and examine factors associated with PrEP indication.

## 2 Methods

### 2.1 Participants and procedures

Between November and December 2017, a national convenience sample of trans MSM in the U.S. (n = 857) was recruited and completed an online one‐time computer‐assisted self‐interview (CASI) survey to characterize HIV sexual risk behaviours and prevention needs, and to assess barriers and facilitators to PrEP. A participatory population methodology was used to conduct this research, working “with” not “on” trans masculine (TM) people 30. The study team engaged a task force of trans MSM identified individuals to provide feedback on the research methods and survey. A variety of recruitment methods were employed, including: peer‐to‐peer networks, dating apps (Grindr), social media, Facebook groups of interest to trans MSM, community linkages such as partnerships with community‐based organizations serving trans MSM, and advertising/outreach at the 2017 Philadelphia Trans Wellness Conference.

Participants were consented via an online form preceding the survey. The Fenway Institute Review Board approved all study procedures. Individuals who met the following criteria were considered eligible for study participation: (a) assigned female sex at birth, (b) current gender identity is on the trans masculine spectrum, (c) age 18 years or older, (d) English‐speaking, and (e) had sex with a cis male partner in the past six months. Participants were compensated with a $10 Amazon gift card upon completion of the survey, which took 30 to 60 minutes to complete on average. The survey was anonymous. To ensure confidentiality, contact information collected for sending gift cards was kept separate from the survey. Email addresses provided on the form were screened for authenticity to identify and exclude survey‐taking “bots” from the data. CAPTCHA technology was also used to prevent fraudulent data entry. The survey enabled HTTP cookies to prevent participants from taking the survey more than once.

### 2.2 Measures

The research team developed a survey with input from a trans MSM Task Force, collecting information on PrEP, demographics, medical gender affirmation, healthcare access and utilization, mental health, substance use, relationships and risk perception, and stigma. Wherever possible, validated survey items and scales were selected from prior research with trans populations and/or MSM PrEP research to ensure comparability across studies.

#### 2.2.1 Descriptive variables: PrEP awareness, uptake and persistence

Participants were asked whether they had ever heard of medication taken to prevent HIV acquisition. Those who had were queried about whether they had ever taken PrEP and were currently taking PrEP.

#### 2.2.2 Outcome variable: PrEP indications, last six months

Sexual behaviours were assessed using questions about sex with cis male partners in the past six months including oral, frontal/vaginal and anal (insertive and receptive) sex both with and without condoms. These measures were adapted from prior trans sexual health research 31. The survey was designed to be gender‐affirming. For example, the terms “front hole” and “frontal sex” were used to describe receptive frontal/vaginal sex. Whether or not survey participants had condomless sex with HIV positive partners, partners of unknown HIV serostatus, and in the context of substance use were assessed. Current PrEP indication guidelines are difficult to apply and may not be appropriate for trans MSM. Future research is especially needed to consider gender of sexual partners in PrEP indications, including transgender partners. For the purpose of this study, and to be as consistent as possible with CDC's guidelines on PrEP indication, only condomless receptive frontal/vaginal and/or anal sex were considered high risk 14. We included receptive frontal/vaginal sex in addition to receptive anal sex due to the potential for many trans MSM to acquire HIV through either or both anatomical sites. We applied PrEP indication guidelines to identify PrEP indicated respondents using algorithms for MSM, heterosexuals and injection drug use (IDU) 14. For IDU, risk of sexual acquisition was required to meet PrEP eligibility criteria. History of any bacterial sexually transmitted infection (STI) diagnosis in the past six months was self‐reported by participants on the survey. Table 1 summarizes the application of these guidelines to trans MSM. If respondents met PrEP indications for one or more of these algorithms they were considered to be PrEP indicated and compared to those with no PrEP indications (binary PrEP indication outcome yes/no).

**Table 1:** Application and operationalization of PrEP indications algorithms^a^

Column 1: men who have sex with men			Column 2: heterosexually active men and women			Column 3: people with injection drug use		
Guideline component	How operationalized	Limitations	Guideline component	How operationalized	Limitations	Guideline component	How operationalized	Limitations
Adult man	Age 18 + required for study participation	–	Adult person	Age 18 + required for study participation	–	Adult person	Age 18 + required for study participation	–
Without acute or established HIV infection	Self‐reported as HIV‐negative or unknown	–	Without acute or established HIV infection	Self‐reported as HIV‐negative or unknown	–	Without acute or established HIV infection	Self‐reported as HIV‐negative or unknown	–
Any male sex partners in past six months	Sex with cis male in last six months was required for study participation	–	Any sex with “opposite sex” partners in past six months	Did not operationalize “opposite sex”	Sex with cis male in last six months was required for study participation	Any injection of drugs not prescribed by a clinician in past six months	Injected drugs to get high in the last six months (does not include testosterone injections)	Unable to evaluate whether or not drugs were clinician prescribed
Not in a monogamous partnership with a recently tested, HIV‐negative man	Self‐reported more than one sexual partner in last six months	Unable to account for “with a recently tested, HIV‐negative” man	Not in a monogamous partnership with a recently tested, HIV‐negative partner	Self‐reported more than one sexual partner in last six months	Unable to account for “with a recently tested, HIV‐negative” partner	–	–	–
Must also have at least one of …			Must also have at least one of…			Must also have at least one of…		
Any anal sex without condoms (receptive or insertive) in past six months	Any condomless receptive anal or frontal/vaginal sex in last six months	One or more partners that recipient had condomless receptive anal or frontal/vaginal sex with in the last six months	Is a man who has sex with both women and men (behaviourally bisexual)	Partners of any gender other than cis males (cis females, trans men, trans women)	Not limited to cis male or cis female	Any sharing of injection drug preparation equipment in past six months	Not asked	Unable to account for sharing of works
A bacterial STI (syphilis, gonorrhoea, or chlamydia) diagnosed or reported in last six months	Self‐reported STI diagnosis of syphilis, gonorrhoea, and/or chlamydia in the last six months		Infrequently uses condoms during sex with one or more partners of unknown HIV status who are known to be at substantial risk of HIV infection (persons with injection drug use or bisexual male partner)	People who had sex without a condom with a partner of unknown HIV status and had a sexual partner who uses injection drugs OR exchanged sex for money, drugs, gifts, or services in the last six months	Unable to account for frequency of condom use or other HIV risk behaviours	Risk of sexual acquisition	See MSM and Heterosexually Active Women and Men Criteria	
			Is in an ongoing sexual relationships with an HIV‐positive partner	Any condomless sex in the last six months (receptive or insertive, anal or frontal/vaginal) with a partner known to have HIV				
			A bacterial STI (syphilis, gonorrhoea, or chlamydia) diagnosed or reported in last six months	Self‐reported STI diagnosis of syphilis, gonorrhoea, or chlamydia in the last six months				

PrEP, pre‐exposure prophylaxis.

Guidelines per Centers for Disease Control and Prevention (CDC): https://www.cdc.gov/hiv/pdf/risk/prep/cdc-hiv-prep-guidelines-2017.pdf.

#### 2.2.3 Statistical predictors

Six blocks of statistical predictors were considered for PrEP indication, grouped according to the following themes: sociodemographics; medical gender affirmation; healthcare access and utilization; mental health and substance use; relationships and risk perception; and stigma.

Sociodemographics: Age was assessed in years and grouped into CDC categories for ages 18 to 24, 25 to 29, 30 to 39, 40 to 60 years 32. Gender identity was assessed as “If you had to select ONE response that best describes your current gender identity today for the purposes of a survey, what would it be?” and coded as binary (male, man, transgender man, female‐to‐male, trans man, man of transgender experience) versus nonbinary (trans masculine, genderqueer, gender‐nonconforming, non‐binary, agender, bigender, other gender). Sexual orientation identity was asked as “Which of the following best describes your sexual identity or orientation today?” To assess the potential independent influence of a gay identity, a variable was coded as gay‐identified (gay, homosexual, same‐gender attraction) versus not gay‐identified (bisexual, queer, pansexual, other). Race was assessed with the question “How do you describe your race or ethnic background? Check all that apply” and coded as white, black or other (Asian, American Indian/Alaskan, Pacific Islander, Multiracial, or other). Ethnicity was coded as Hispanic/Latinx or non‐Hispanic/Latinx. Educational attainment was categorized as high school or less; trade school, some undergraduate, or associate's degree; undergraduate degree; some graduate school; or graduate degree. Urbanicity (urban, rural) and U.S. region (Northeast, Midwest, South, West) were coded from self‐reported zip code.

Medical gender affirmation: A series of items from prior research with trans masculine adults assessed medical gender affirmation, including lifetime testosterone use, top surgery and bottom surgery 31. Participants who indicated top surgery procedures (e.g. mastectomy, chest reduction) were considered to have had top surgery; those reporting lower surgery (e.g. phalloplasty, metoidioplasty) were coded as having had lower surgery.

Healthcare access and utilization: Participants were asked about what type of insurance they have. Health insurance status was operationalized as insured versus not insured. HIV prevention services were assessed including history of HIV testing and accessing HIV programmes. Having ever tested for HIV (yes, no) and ever accessed HIV programmes (yes, no) were coded.

Mental health and substance use: The validated Kessler 6‐item (K6) scale measured psychological distress in the last 30 days 33. Questions asked participants to rate how often they felt different symptoms, for example “nervous,” “hopeless” and “worthless.” Responses ranged from 0 (none of the time) to 4 (most of the time) yielding a score of 0 to 24 (Cronbach's alpha in this sample = 0.88). K6 scores were grouped by quartile distribution. Hazardous drinking was measured by AUDIT‐C 34, 35, a three‐item validated screener ranging from 0 to 12. A score of four or more or was used as a cut‐off for hazardous drinking (yes, no) 34, 35. Non‐marijuana illicit poly‐drug use in the last six months was operationalized as reporting three or more of the following 10 illicit drugs (cocaine, crack cocaine, club drugs, heroin, methamphetamine, poppers, hallucinogens, downers, painkillers and uppers) consistent with the International Classification of Diseases, 10^th^ Revision, Clinical Modification 36.

Relationships and risk perception: Participants were asked a series of relationship questions including whether they were currently polyamorous, defined as having more than one intimate relationship at a time with the knowledge and consent of everyone involved (yes, no), had met a sexual partner online in the last six months (yes, no), and had engaged in sex exclusively with cis men in the last six months (yes, no). Participants were asked about their perceived HIV risk in the last six months on a scale from 0 (least risky) to 10 (most risky). Perceived HIV risk was grouped by quartile distribution. Participants reported total number of sexual partners in the last six months. Number of partners was categorized in quartiles.

Stigma: Cis male partner stigma in the past six months was assessed using a four‐item scale previously developed for young adult trans MSM 8. Sample items “I have been mis‐pronouned/misgendered during or after sex” and “I have crossed boundaries…to validate my gender identity or expression in the sexual encounter.” Response options were on a Likert scale from “Never” to “Many times” yielding a score ranging from 0 to 12. Items were summed so that higher scores indicated more stigma from cis male partners (Cronbach's alpha = 0.83). Internalized stigma was measured using five items on a Likert scale ranging from “Strongly Agree” to “Strongly Disagree” (scores 0 to 30). Sample items: “I wish I were not transgender or gender‐nonconforming” and “I dislike myself for being gay/bisexual/queer/attracted to men.” Partner stigma and internalized stigma scores were each grouped by quartile distribution.

### 2.3 Data analysis

There was a moderate magnitude of missing data (14.7% of all item‐level data points). Of the 857 trans MSM survey participants, 52% (N = 447) had non‐missing data on all 123 survey items used to measure PrEP‐indication and the statistical predictors. Therefore, to avoid selection bias induced by naïve complete‐case analysis, all analyses were conducted on multiply imputed data (M = 5) obtained via Multiple Imputation by Chained Equations (MICE) with random forests in R 37, 38. Pooled estimates were obtained by combining estimates from each dataset into a single parameter estimate, using appropriate methods (PROC MIANALYZE in SAS).

Since the outcome of interest is PrEP indications, we excluded HIV positive participants (N = 14, 1.6%) for a final N of 843. Descriptive statistics were obtained to characterize the distribution of variables in the sample overall, and by PrEP indications (outcome of interest). Bivariate analyses were conducted using logistic regression. Variables assessed were age, binary gender identity, gay sexual orientation, race, ethnicity, education, urbanicity, region, testosterone use, top surgery, bottom surgery, insurance status, HIV testing, access to HIV programmes, depression, hazardous alcohol use, poly‐drug use, polyamorous relationship status, having met a sex partner online, sex only with cis men, perceived HIV risk, number of sexual partners, partner stigma and internalized stigma. For multivariable models, PrEP indication was modelled in a block‐wise fashion using each of the six groups of statistical predictors (sociodemographics, medical gender affirmation, healthcare access and utilization, mental health and substance use, relationships and risk perception, and stigma) as blocks. All variables reaching <0.05 significance in block models were included in a final multivariable model. The final model controlled for all sociodemographic variables regardless of their statistical significance (age, binary gender identity, gay sexual orientation, race, ethnicity, education, urbanicity and region). Analyses were conducted using SAS 9.4.

## 3 Results

### 3.1 Sample characteristics

Descriptive characteristics of the study population (n = 857) are presented in Table 2 (column 3). The majority of sample (65.3%) was under the age of 30 years, 69.7% were White, 4.8% were Black, and 77.9% non‐Hispanic/Latinx. The majority (71.6%) identified as a binary gender identity (e.g. man, male, transgender man). Nearly one‐third (32.6%) identified their sexual orientation as gay. The majority (62.6%) had less than or equal to some undergraduate education. The sample predominantly (64.6%) lived in urban areas and was distributed widely in the U.S.: Northeast (19.5%), Midwest (14.2%), South (24.4%) and West (22.6%).

**Table 2:** Overall descriptive characteristics of the study sample of trans MSM in the U.S. (N = 857) by PrEP indications: PrEP Indications (N = 465) and no PrEP indications (N = 378)

	Column 1	Column 2	Column 3
	PrEP indications^a^ (N = 465)	No PrEP indications^a^ (N = 378)	Total sample (N = 857)
	55.2%	44.8%	
	**%**	**%**	**%**
Sociodemographics			
Age in years			
18 to 24	30.6	34.1	32.0
25 to 29	31.9	34.8	33.3
30 to 39	30.8	25.9	28.5
40 to 60	6.7	5.2	6.2
Binary gender identity			
Yes	86.3	87.6	71.6
No	13.7	12.4	28.4
Gay sexual orientation identity			
Yes	31.5	34.4	32.6
No	68.5	65.6	67.4
Race			
White	69.6	69.7	69.7
Black	5.3	4.2	4.8
Other	25.1	26.1	25.5
Hispanic/latinx ethnicity			
Yes	23.8	19.1	22.1
No	76.2	80.9	77.9
Educational attainment			
High school or less	14.7	12.9	13.9
Trade school, some undergraduate, or associate's degree	50.4	46.6	48.7
Undergraduate degree	18.4	23.4	20.8
Some graduate school	4.9	8.4	6.5
Graduate degree	11.6	8.7	10.1
Urban geographic locale			
Yes	64.0	65.8	64.6
No	14.5	16.1	15.3
Other/unknown geography	21.5	18.2	20.1
Region			
Northeast	19.0	20.3	19.5
Midwest	13.3	15.8	14.2
South	24.0	25.1	24.4
West	23.2	21.3	22.6
Other/unknown geography	20.5	17.5	19.3
Medical gender affirmation			
Testosterone use			
Yes	82.0	83.2	82.7
No	18.0	16.8	17.3
Top surgery			
Yes	48.0	49.8	51.4
No	52.0	50.2	48.6
Bottom surgery			
Yes	17.9	22.6	20.7
No	82.1	77.4	79.3
Healthcare access and utilization			
Health insurance			
Yes	90.6	94.1	92.3
No	9.4	5.9	7.7
Ever been tested for HIV			
Yes	80.0	79.4	79.8
No	20.0	20.6	20.2
Ever accessed HIV programmes			
Yes	39.4	33.4	37.1
No	60.6	66.6	62.9
HIV status based on self‐reported most recent HIV test			
HIV positive	0.0	0.0	1.6
HIV negative or unknown	0.0	0.0	98.4
Self‐reported bacterial STI diagnosis in past six months			
Yes	26.3	3.7	16.4
No	73.7	96.3	83.6
Mental health			
Psychological distress			
No distress	15.2	20.1	17.5
Low levels of distress	32.4	38.7	34.8
Moderate levels of distress	28.7	26.1	27.8
High levels of distress	23.7	15.1	19.9
Heavy/hazardous drinking			
Yes	65.1	52.4	59.5
No	34.9	47.6	40.5
Polydrug use (use of three or more non‐marijuana illicit drugs)			
Yes	25.4	12.1	20.0
No	74.6	87.9	80.0
Relationships and risk perception			
Polyamorous relationship			
Yes	65.4	46.5	57.2
No	34.6	53.5	42.8
Met a sex partner online, last six months			
Yes	37.5	52.5	44.5
No	62.5	47.5	55.5
Sex with cisgender man/men only, last six months			
Yes	30.4	55.1	41.5
No	69.6	44.9	58.5
Perceived HIV risk			
Low risk	16.3	37.7	26.0
Moderate‐low risk	30.2	30.0	29.8
Moderate‐high risk	20.5	18.0	19.1
High risk	33.0	14.3	25.1
Stigma			
Partner stigma			
No stigma	13.8	32.7	22.0
Low stigma	32.2	34.5	33.0
Moderate stigma	34.5	25.8	31.0
High stigma	19.5	7.0	14.0
Internalized stigma			
Very low stigma	25.3	33.4	28.7
Low stigma	27.2	31.8	29.0
Moderate stigma	26.0	20.3	23.7
High stigma	21.5	14.5	18.6

Those who self‐reported as HIV positive (n = 14) were not included in columns 1 and 2. PrEP, pre‐exposure prophylaxis.

PrEP Indications variable (column 1: PrEP indications, and column 2: no PrEP indications) exclude respondents that disclosed HIV positive status (n = 14). The denominator for these columns is n = 843.

For medical gender affirmation, 82.7% had taken testosterone, 51.4% had top surgery, and 20.7% had bottom surgery. In terms of healthcare access, 92.3% were insured, 79.8% had ever been tested for HIV and 37.1% had accessed HIV prevention programming. Levels of psychological distress were high. Overall, 59.5% met criteria for heavy/hazardous drinking and 20.0% endorsed poly‐drug use. The majority (57.2%) reported being in a polyamorous relationship. Over one‐third (44.5%) had met a sex partner online in the last six months. 41.5% exclusively reported sex with cis men in the last six months; 58.5% reported sex with a cis male partner (criteria for study eligibility) and with partners of other genders. Exposure to cis male partner stigma and internalized stigma was high.

### 3.2 PrEP awareness, uptake and persistence

Overall, 84.1% of the sample had heard of PrEP to prevent HIV acquisition. Of these, 33.3% reported lifetime PrEP use: 21.8% currently taking PrEP and 11.5% having stopped taking PrEP. Among participants currently taking PrEP, 64.8% met indications for PrEP. Of those who had stopped taking PrEP, 75.9% met PrEP indications.

### 3.3 PrEP indications, last six months

**Figure 1:**
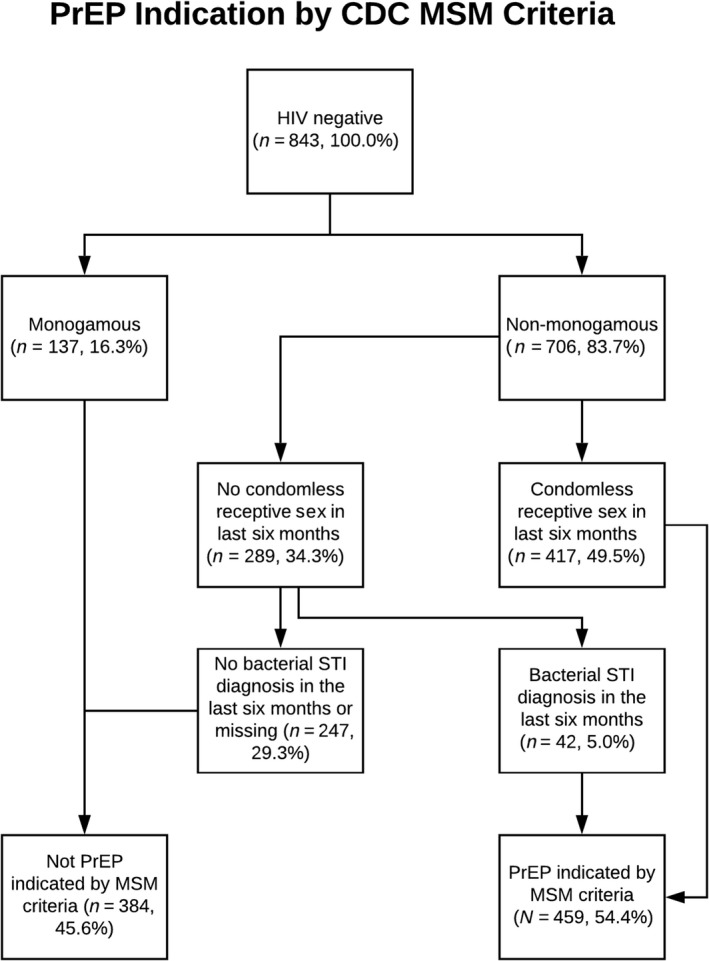
PrEP Indication by CDC MSM Criteria PrEP, pre‐exposure prophylaxis; CDC, Centers for Disease Control and Prevention; MSM, men who have sex with men.

**Figure 2:**
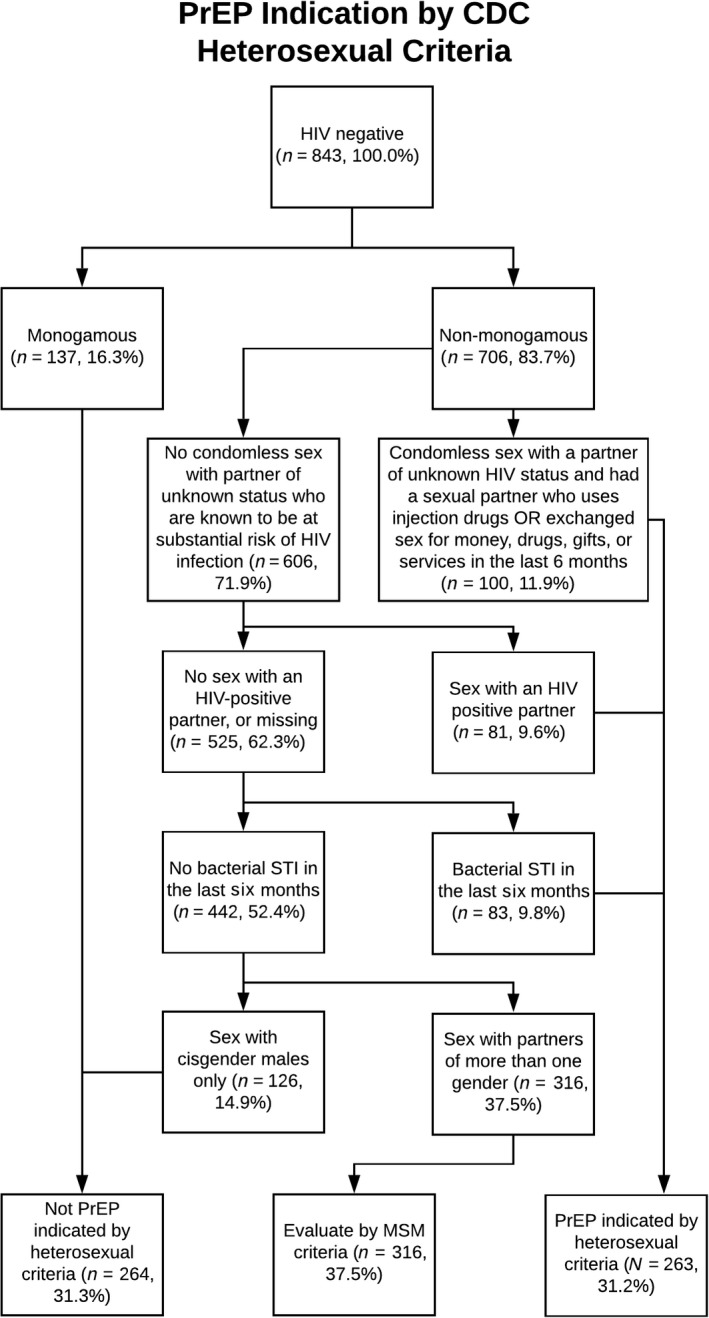
PrEP Indication by CDC Heterosexual Criteria PrEP, pre‐exposure prophylaxis; CDC, Centers for Disease Control and Prevention

**Figure 3:**
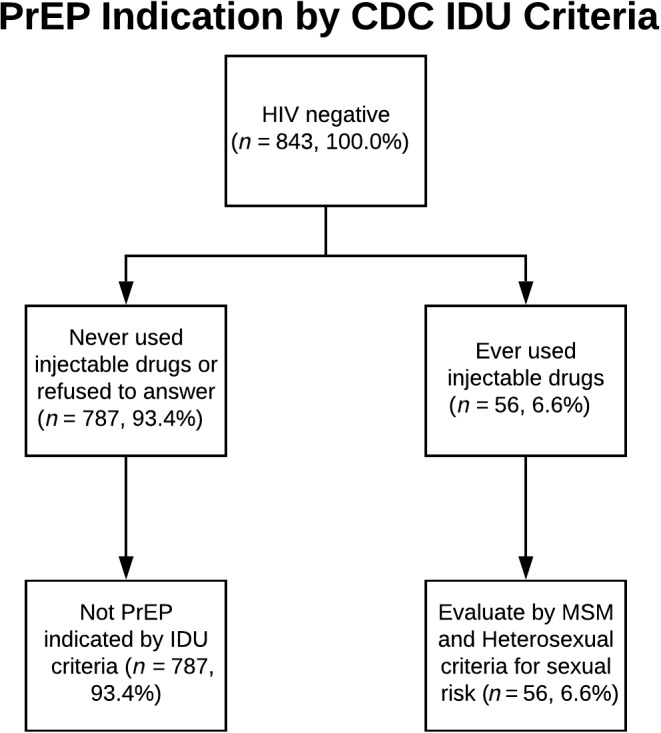
PrEP Indication by CDC IDU Criteria PrEP, pre‐exposure prophylaxis; CDC, Centers for Disease Control and Prevention

Three adapted CDC algorithms for PrEP indication were applied to trans MSM respondents (Table 1, columns 1 to 3): 54.4% of trans MSM were PrEP indicated based on MSM criteria (Figure 1), 31.2% based on the heterosexual guideline (Figure 2), and 6.6% according to IDU (Figure 3).

**Figure 4:**
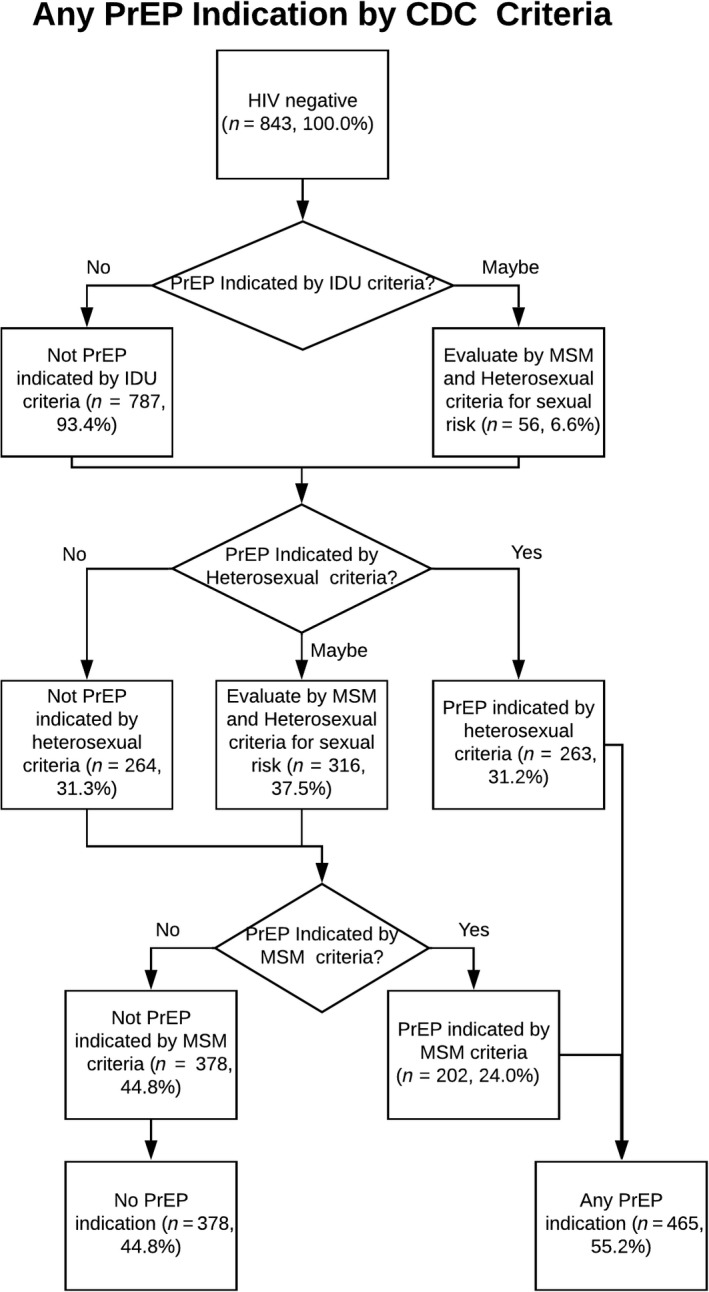
PrEP Indication by Any CDC Criteria PrEP, pre‐exposure prophylaxis; CDC, Centers for Disease Control and Prevention

Overall, 55.2% of trans MSM respondents had PrEP indication per CDC guidelines (Figure 4).

Sample characteristics by PrEP indications (n = 465) and no PrEP indications (n = 378) are presented in Table 2 (columns 1 and 2).

### 3.4 Regression models of PrEP indication

Table 3 displays results from logistic regression models regressing PrEP indication (yes/no) on sociodemographic, medical gender affirmation, healthcare access and utilization, mental health, substance use, relationships and perceived risk and stigma variables. Bivariate analyses are shown in Table 3, Column 1.

**Table 3:** Bivariate, block and multivariable logistic regression models of PrEP indication among HIV‐negative trans MSM in the U.S. (N = 843)

	Column 1: bivariate models			Column 2: block models			Column 3: multivariable model		
	OR	95% CI	*p*‐value	aOR	95% CI	*p*‐value	aOR	95% CI	*p*‐value
Sociodemographics									
Age in years									
18 to 24	REF			REF			REF		
25 to 29	1.03	0.71, 1.47	0.89	1.14	0.77, 1.69	0.52	0.93	0.55, 1.57	0.79
30 to 39	1.33	0.90, 1.96	0.15	1.41	0.90, 2.20	0.14	1.25	0.76, 2.06	0.37
40 to 60	1.46	0.69, 3.11	0.32	1.54	0.65, 3.62	0.32	1.26	0. 42, 3.87	0.66
Binary gender identity									
Yes	0.89	0.57, 1.39	0.61	0.84	0.53, 1.34	0.47	0.85	0.48, 1.51	0.58
No	REF			REF			REF		
Gay sexual orientation identity									
Yes	REF			REF			REF		
No	1.14	0.84, 1.54	0.41	1.21	0.86, 1.72	0.28	0.93	0.61, 1.41	0.73
Race									
White	REF			REF			REF		
Black	1.26	0.63, 2.54	0.51	1.22	0.59, 2.52	0.59	0.79	0.35, 1.78	0.68
Other race or multiracial	0.96	0.67, 1.37	0.83	0.87	0.61, 1.26	0.46	0.84	0.54, 1.32	0.46
Hispanic/latinx ethnicity									
Yes	1.32	0.82, 2.13	0.24	1.32	0.79, 2.21	0.27	0.83	0.45, 1.51	0.52
No	REF			REF			REF		
Educational attainment									
High school or less	REF			REF			REF		
Trade school, some undergraduate, or associate's degree	0.95	0.55, 1.66	0.86	0.98	0.54, 1.77	0.94	0.60	0.20, 1.83	0.34
Undergraduate degree	0.69	0.35, 1.40	0.29	0.70	0.31, 1.56	0.35	0.91	0.42, 1.93	0.78
Some graduate school	0.52	0.25, 1.05	0.07	0.50	0.24, 1.04	0.06	0.43	0.17, 1.08	0.07
Graduate degree	1.17	0.61, 2.25	0.63	1.07	0.51, 2.28	0.85	1.08	0.40, 2.92	0.87
Urban geographic locale									
Urban	REF			REF			REF		
Rural	0.93	0.58, 1.48	0.75	0.93	0.56, 1.53	0.76	0.94	0.51, 1.75	0.85
Other/unknown geography	1.22	0.78, 1.91	0.37	1.70	0.28, 10.28	0.56	1.45	0.17, 12.60	0.73
Region									
Northeast	1.11	0.68, 1.81	0.69	1.05	0.63, 1.73	0.86	1.18	0.65, 2.17	0.58
Midwest	REF			REF			REF		
South	1.13	0.65, 1.98	0.66	1.07	0.60, 1.90	0.81	1.06	0.53, 2.15	0.86
West	1.29	0.80, 2.06	0.29	1.20	0.74, 1.96	0.46	1.29	0.74, 2.27	0.37
Other/unknown geography	1.39	0.82, 2.35	0.22	0.75	0.11, 5.17	0.77	0.77	0.08, 7.75	0.82
Medical gender affirmation									
Testosterone use									
Yes	0.92	0.60, 1.41	0.70	0.92	0.58, 1.47	0.72	–	–	–
No	REF			REF			–	–	–
Top surgery									
Yes	0.93	0.70, 1.24	0.63	0.98	0.70, 1.37	0.91	–	–	–
No	REF			REF			–	–	–
Bottom surgery									
Yes	0.74	0.46, 1.20	0.22	0.75	0.45, 1.24	0.24	–	–	–
No	REF			REF			–	–	–
Healthcare access and utilization									
Health insurance									
Yes	REF			REF			–	–	–
No	1.64	0.96, 2.81	0.07	1.63	0.95, 2.79	0.08	–	–	–
Ever been tested for HIV									
Yes	1.04	0.72, 1.52	0.83	0.99	0.68, 1.45	0.97	–	–	–
No	REF			REF			–	–	–
Ever accessed HIV programmes									
Yes	1.30	0.96, 1.76	0.09	1.29	0.95, 1.76	0.10	–	–	–
No	REF			REF			–	–	–
Mental health and substance use									
Psychological distress, last 30 days									
No distress	REF			REF			REF		
Low levels of distress	1.11	0.64, 1.90	0.70	1.08	0.63, 1.87	0.76	0.99	0.43, 2.27	0.98
Moderate levels of distress	1.45	0.85, 2.47	0.17	1.33	0.79, 2.24	0.28	1.18	0.52, 2.67	0.67
High levels of distress	**2.07**	**1.17, 3.67**	**0.01**	**1.80**	**1.02, 3.18**	**0.04**	1.45	0.61, 3.44	0.37
Heavy/hazardous drinking									
Yes	**1.70**	**1.20, 2.40**	**<0.01**	**1.48**	**1.03, 2.14**	**0.03**	1.19	0.75, 1.88	0.44
No	REF			REF			REF		
Polydrug use, last six months^a^									
Yes	**2.49 **	**1.58, 3.91**	**<0.01**	**2.04**	**1.28, 3.25**	**<0.01**	1.19	0.70, 2.03	0.51
No	REF			REF			REF	–	–
Relationships and risk perception									
Polyamorous relationship									
Yes	**2.17**	**1.51, 3.13**	**<0.01**	1.35	0.90, 2.00	0.14	–	–	–
No	REF			REF			–	–	–
Met a sex partner online, last six months									
Yes	**0.54**	**0.37, 0.80**	**<0.01**	**0.42**	**0.26, 0.68**	**<0.01**	**0.44**	**0.28, 0.70**	**<0.01**
No	REF			REF			REF		
Sex with cisgender man/men only, last six months									
Yes	REF			REF			REF		
No	**2.81**	**2.05, 3.85**	**<0.01**	**1.84**	**1.21, 2.78**	**0.01**	**1.84**	**1.13, 3.00**	**0.02**
Perceived HIV risk, last six months									
Low risk	REF			REF			REF		
Moderate‐low risk	**2.32**	**1.55, 3.45**	**<0.01**	**1.84**	**1.19, 2.83**	**0.01**	**1.90**	**1.22, 2.97**	**<0.01**
Moderate‐high risk	**2.63**	**1.67, 4.14**	**<0.01**	**2.49**	**1.46, 4.25**	**<0.01**	**2.54**	**1.47, 4.37**	**<0.01**
High risk	**5.32**	**3.21, 8.82**	**<0.01**	**4.63**	**2.50, 8.58**	**<0.01**	**4.17**	**2.14, 8.12**	**<0.01**
Number of partners in the last six months									
≤2 partners	REF			REF			REF		
3 partners	**3.02**	**1.89, 4.82**	**<0.01**	**2.47**	**1.48, 4.13**	**<0.01**	**2.70**	**1.50, 4.86**	**<0.01**
4 to 6 partners	**5.30**	**3.37, 8.34**	**<0.01**	**3.59**	**2.11, 6.12**	**<0.01**	**3.68**	**2.06, 6.59**	**<0.01**
>6 partners	**10.20**	**6.10, 17.07**	**<0.01**	**6.78**	**3.48, 13.22**	**<0.01**	**7.13**	**3.34, 15.20**	**<0.01**
Stigma									
Partner stigma, last six months									
No stigma	REF			REF			REF		
Low stigma	**2.22**	**1.35, 3.66**	**<0.01**	**2.15**	**1.30, 3.56**	**<0.01**	1.59	0.89, 2.86	0.11
Moderate stigma	**3.18**	**1.86, 5.43**	**<0.01**	**2.96**	**1.70, 5.14**	**<0.01**	1.50	0.85, 2.63	0.16
High stigma	**6.62**	**3.72, 11.78**	**<0.01**	**6.03**	**3.38, 10.77**	**<0.01**	**2.30**	**1.12, 4.73**	**0.02**
Internalized stigma									
Very low stigma	REF			REF			–	–	–
Low stigma	1.13	0.76, 1.67	0.56	0.99	0.65, 1.49	0.95	–	–	–
Moderate stigma	**1.69**	**1.08, 2.64**	**0.02**	1.33	0.83, 2.12	0.23	–	–	–
High stigma	**1.95**	**1.19, 3.20**	**0.01**	1.34	0.78, 2.25	0.28	–	–	–

95% CI, 95% confidence interval; OR, odds ratio; MSM, men who have sex with men; PrEP, pre‐exposure prophylaxis.

a Polydrug Use: Operationalized as use of three or more non‐marijuana illicit drugs. Bold indicates statistical significance *p* < 0.05.

In block models (Table 3, Column 2), statistically significant increases in HIV risk were found for the following variables: sociodemographics (identifying as gay vs. not), mental health and substance use (high psychological distress, hazardous alcohol use and non‐marijuana illicit poly‐drug use), relationships and risk perception (polyamorous relationship, not having sex only with a cis male partner in the last six months, higher perceived HIV risk, higher number of sexual partners), and stigma (higher levels of stigma by cis male sexual partners). There were no significant differences in PrEP indication found for variables in medical gender affirmation or healthcare access and utilization blocks.

In a final multivariable model (Table 3, Column 3), factors statistically significantly associated with increased odds of PrEP indication and HIV risk in trans MSM were not meeting a sex partner online, not having sex only with cisgender men, higher perceived HIV risk, greater number of sexual partners and high partner stigma compared to no partner stigma.

## 4 Discussion

While the U.S. Food and Drug Administration approved the first oral drug for PrEP use in 2012, the current study is the first to our knowledge to quantitatively investigate PrEP indications in trans MSM nationally. The majority of this sample of trans MSM in the U.S. had heard of PrEP to prevent HIV infection, but PrEP uptake was low despite that indications for PrEP were high. Approximately five of ten respondents were candidates who could benefit from PrEP to prevent HIV acquisition. Additionally, the current study found that the proportion of trans MSM who were PrEP indicated differed based on the CDC criteria applied. Merging CDC algorithms resulted in prevalence of PrEP indications similar to that found in presumably cis MSM sampled online 39. These findings suggest the need for additional guidance to inform PrEP continuum and care delivery for trans MSM, including algorithms to identify PrEP candidates for HIV prevention service delivery. PrEP indication measures are largely determined by HIV risk and some HIV risks may pertain to trans MSM specifically. Ongoing research is needed to inform national guidelines for PrEP indication for trans people, including for trans MSM at‐risk of HIV acquisition. For the most part, HIV biobehavioural prevention interventions have not been designed with trans MSM in mind. For example, trans MSM are excluded from participating in clinical PrEP trials; being assigned female at birth was exclusionary for large scale clinical trial iPrEx 12. Results from this study support the full inclusion of trans MSM in HIV biobehavioural prevention efforts. Additional data regarding feasibility of PrEP implementation models, barriers/facilitators to PrEP adherence and acceptability of PrEP modalities (e.g. injectables) among trans MSM will be important next steps for research.

Higher perceived HIV risk was found to be associated with increased odds of PrEP indication among trans MSM. Prior research on perceived versus actual risk in trans MSM have been mixed, with some studies finding concordance and other others not 6, 7. The congruency of high perceived risk and PrEP indications in this sample may be related to study eligibility criteria which was limited to trans MSM who had sex with cis man in the last six months. In research with cis MSM sampled online, higher risk perception has been associated with reduced odds of condomless sex suggesting a discordance of perceived risk and reported sexual behaviour 26. More research is needed to understand HIV risk perceptions and its relation to PrEP indication in MSM, trans and cis alike. Interventions that include PrEP screening and delivery of PrEP services may benefit from the inclusion of risk‐related messaging for trans MSM.

Other risks specific to trans MSM emerged, specifically stigma from cis male sex partners. Reports of high partner stigma, compared to no partner stigma, were associated with increased odds of PrEP indication. Trans MSM health risks are situated with the context of societal stigma and socialization, including from within sexual and gender minority communities 19. Biomedical HIV prevention interventions, including those delivering PrEP, need to address and be responsive to these situated vulnerabilities in order to be culturally relevant for trans MSM.

All trans MSM sampled reported sex with a cis male (this was a study inclusion criterion); however, trans MSM who reported not having sex exclusively with cis men had elevated odds of PrEP indication. Said differently, those trans MSM who reported sex with a cis male(s) and one or more partner(s) of other gender(s) had higher odds of PrEP indication than trans MSM who exclusively reported sex with cis males. This finding remained significant after adjusting for number of sexual partners, and having a higher number of sexual partners was associated with increased probability of PrEP indications. Approximately 40% of the sample reported sex with cis men only in the last six months. Prior studies demonstrate that monosexuality (i.e. having romantic or sexual attractions to members of one sex or gender only) is less common than non‐monosexuality (e.g. bisexual, pansexual) in trans MSM 3, 19, 23. In the current sample, approximately one‐third of trans MSM identified their sexual orientation as gay; the majority were non‐monosexual (e.g. queer) and had sexual partners of diverse genders. The CDC criteria linked to the language of “behaviourally bisexual” is difficult in a study population with a high proportion of non‐monosexuals. Additional study of sexual partnerships and network characteristics of trans MSM is warranted, including behavioural risks by sexual orientation identity and genders of sexual partners.

The strengths of this study include being a large sample of trans MSM nationally, utilizing community‐engaged research methods, having a gender‐affirmative assessment of PrEP, and understanding more about an at‐risk population about which little is known. This study has several limitations. In several instances, applying CDC guidelines required interpretation due to not having the exact variable needed. In other cases, CDC guidelines were interpreted for trans MSM specifically. For example, the condomless receptive anal sex criterion for MSM indication was extended to include condomless receptive frontal/vaginal and anal sex to account for the fact that each confer risk for HIV acquisition for trans MSM. This was a convenience sample of trans MSM that is most likely not representative of the trans MSM population in its entirety. However, findings are generally consistent with existing HIV‐related studies in other high income settings 6, 7. Respondents were recruited from a variety of sources, including social media, peer networks, and dating apps. This may have introduced selection bias in that trans MSM who heard of the study and/or opted to participate may be different in HIV risk and associated characteristics than those who did not participate. For example, our sample may be highly engaged and active online trans MSM networks, which may increase exposure to PrEP messaging and could explain the prevalence of PrEP uptake found. Data were self‐report and no biomarker data were available to confirm HIV‐uninfected serostatus. This was a cross‐sectional survey of a single time‐point. Missing data were handled using imputation methods flexible and robust under multiple scenarios of missingness; however, it assumed data were missing at random. Future research is recommended that can overcome these limitations, including interventional research to link trans MSM who are PrEP indicated to biobehavioural prevention including PrEP services.

## 5 Conclusions

This study fills an important gap. Limited data are available characterizing HIV risk and prevention needs for trans MSM, especially PrEP awareness, uptake and indications. This study increases the visibility of trans MSM in HIV prevention research, demonstrates a need for PrEP service delivery to this subgroup of MSM, and highlights the necessity for future research, including longitudinal studies to assess PrEP indication over time, and to monitor PrEP uptake, persistence, and acceptability and feasibility of biomedical prevention in trans MSM. Additional guidance is needed for trans MSM algorithms to identify PrEP candidates for HIV prevention services. Findings from this study dispel the myth that trans MSM will not benefit from access to and uptake of PrEP. Public health interventions and programmes are needed to reach trans MSM that address and attend to general MSM risk factors as well as to vulnerabilities specific to trans MSM, including the context of stigma from cis male sexual partners.

## Competing interests

This study was supported by an unrestricted grant from Gilead Sciences to Dr. Kenneth H. Mayer. Dr. Gal Meyer is an employee of Gilead Sciences.

## Authors’ contributions

SLR and KHM designed the research study, oversaw implementation, analyses and interpretation of findings, drafted sections of the manuscript and critically reviewed the manuscript prior to submission. CSM and AS implemented analyses, interpreted results, drafted sections of the manuscript and critically reviewed the manuscript. AA and DJP implemented data collection, conducted the literature review, drafted sections of the manuscript and critically reviewed the manuscript. GM critically reviewed the manuscript prior to submission. All authors have made substantial contributions qualifying them for authorship.

## Abbreviations

CDC: Centers for Disease Control and Prevention
IDU: injection drug use
MSM: men who have sex with men
PrEP: pre‐exposure prophylaxis
Trans MSM: trans masculine people who have sex with cis males
TM: trans masculine
